# Recent Updates and Advances in the Association Between Vitamin D Deficiency and Risk of Thrombotic Disease

**DOI:** 10.3390/nu17010090

**Published:** 2024-12-29

**Authors:** Amirhossein Faghih Ojaroodi, Fatemeh Jafarnezhad, Zahra Eskandari, Shayan Keramat, Agata Stanek

**Affiliations:** 1 Hematology and Transfusion Medicine Ward, Department of Medical Biochemistry, Faculty of Medicine, Tabriz University of Medical Sciences, Tabriz 5166616471, Iran; amirfaghih1103@gmail.com; 2 Department of Hematology, Faculty of Medicine, Ferdowsi University of Mashhad, Mashhad 9177899191, Iran; 3 Department of Hematology, Faculty of Allied Medicine, Bushehr University of Medical Sciences, Bushehr 7518759577, Iran; zhraaeskndrii@gmail.com; 4 VAS-European Independent Foundation in Angiology/Vascular Medicine, Via GB Grassi 74, 20157 Milan, Italy; shayan.sk1993@gmail.com; 5 Support Association of Patients of Buerger’s Disease, Buerger’s Disease NGO, Mashhad 9183785195, Iran; 6 Department of Internal Medicine, Metabolic Diseases and Angiology, Faculty of Health Sciences in Katowice, Medical University of Silesia, Ziołowa 45/47, 40-635 Katowice, Poland

**Keywords:** vitamin D, thrombotic diseases, endothelial cells, oxidative stress, inflammatory pathways

## Abstract

Vitamin D (VD) is a vital lipophilic secosteroid hormone known for its essential role in maintaining skeletal health and regulating calcium and phosphate metabolism. Recent evidence has begun to illuminate its significance beyond bone health, particularly in relation to thrombosis—a condition characterized by blood clot formation within the vascular system that can lead to serious cardiovascular events such as myocardial infarction and stroke. VD deficiency, defined as a plasma 25-hydroxyVD level below 25 nmol/L, affects a substantial portion of the global population, with prevalence rates ranging from 8% to 18%. This study systematically explores the relationships between VD levels and the risk of thrombosis, investigating the underlying mechanisms including VD’s anticoagulant properties, influence on inflammatory pathways, and interactions with endothelial cells. Epidemiological data suggest that low serum levels of VD correlate with an increased risk of venous thromboembolism (VTE), although the reported findings remain inconsistent. Mechanisms that potentially link VD to thrombotic risk include modulation of thrombomodulin and tissue factor expression, as well as enhancement of anti-inflammatory cytokines. Given the prevalence of VD insufficiency, particularly among populations with limited exposure to sunlight, this research highlights the urgent need for strategies to increase VD levels through dietary modifications and supplementation in order to prevent thrombotic events.

## 1. Introduction

Vitamin D (VD) is a lipophilic secosteroid hormone that is crucial for various physiological functions and is predominantly recognized for its role in maintaining skeletal health by regulating calcium and phosphate metabolism [1,2]. The prevalence of VD deficiency—defined as a plasma level of 25-hydroxyVitamin D (25(OH)D) below 25 and 50 nmol/L—is highly prevalent, with an incidence of approximately 8 to 18% and 24 to 49% worldwide depending on world region. The prevalence slightly decreased from 2000–2010 to 2011–2022, but it was still at a high level [3,4,5].

Thrombosis, characterized by the formation of a blood clot (thrombus) within the vascular system, can occur in the arteries or veins [1].

Arterial thrombosis is commonly associated with myocardial infarction and stroke, while venous thrombosis manifests as venous thromboembolism (VTE), including deep vein thrombosis (DVT) and pulmonary embolism (PE) [6,7,8]. The latter conditions are significant contributors to cardiovascular (CV) morbidity and mortality, affecting approximately 1–2 per 1000 people, underscoring the importance of understanding their risk factors [2,6].

Studies have indicated that patients with CV conditions frequently exhibit low levels of 25(OH)D, while increased VD levels correlate with a lower risk of CVD [9,10]. Additionally, the active variant of Vitamin D—1,25-dihydroxy VD—has shown the ability to inhibit blood coagulation, prompting inquiries into its possible protective function against thrombosis [1,2].

Additionally, VD is thought to possess antithrombotic effects by inhibiting the coagulation cascade. The primary proposed mechanisms for the antithrombotic actions of VD include the “up-regulation of thrombomodulin” and “down-regulation of tissue factor” (TF) [11,12]. Furthermore, VD may enhance the levels of anti-inflammatory cytokine IL10 [13]. Inflammation is recognized as a significant factor in coagulation, with high-sensitivity C-reactive protein (hs-CRP) identified as the principal inflammatory cytokine in this context [14].

The aim of this study is to systematically investigate the correlation between VD level and thrombosis risk, explore the underlying mechanisms that can contribute to any observed associations, and clarify the potential role of VD in thrombosis.

## 2. The Role of Vitamin D and Its Relationship with Thrombosis

### 2.1. Vitamin D in the Blood Clotting Process

Maintaining the balance between clot formation and inhibition is a dynamic and well-calculated process called homeostasis.

Factors such as fibrinogen, von Willebrand factor (VWF), tissue factor (TF), prothrombin, collagen, platelets (and their activating factor), and endothelium cause thrombosis, while factors such as antithrombin, heparin, plasminogen (and its activating factor), thrombomodulin, and proteins C and S prevent the formation of clots and thrombosis. Meanwhile, the effects of VD on thrombotic factors and its inhibitory factors have been well identified in previous studies. The association between low amounts of VD and the development of DVT, as well as the reduction in the risk of unprovoked VTE with the use of VD supplements, have been shown in recent studies, and it has been stated that the risks of DVT and VTE can be mitigated through the treatment of VD deficiency [1,15,16].

The antithrombotic effects of VD may be mediated through the VD receptor (VDR). In mice without VDR, platelet aggregation along with TF increased, while antithrombin and thrombomodulin gene expression decreased, collectively indicating the role of VDR in the antithrombotic process. Recent studies have also shown that various forms of vitamin D reduce synthetic TF analogs and additionally increase thrombomodulin [1].

The increased expression of thrombomodulin and its mRNA is due to increased transcription and not to mRNA stability, with this increased transcription mediated by the VD analogs. KY3 is a potent VD analog whose effect on thrombomodulin mRNA may be due to its high affinity for the VD receptor. VD analogs increase thrombomodulin, and in short, VD analogs can be used as antithrombotic agents in the treatment of inflammatory and atherosclerotic diseases because they reduce TF expression and increase thrombomodulin expression [1,17].

The critical roles of VD and VDR in the regulation of antithrombin transcription have also been revealed in a study that involved disrupting regulatory elements of the VDR promoter region of the gene, suggesting that defects in the VD receptor contribute to antithrombin deficiency and increased risk of thrombosis; furthermore, it was reported that VDR controls the SERPINCI gene, which encodes antithrombin [1,18].

A positive correlation has been reported between the levels of VD and tissue factor pathway inhibitor (TFPI), which is an anticoagulant protein that inhibits coagulation by binding to its complex—namely, factor 7 and factor 10—and TF [15]. Furthermore, the relationship between VD levels above 20 ng/mL and increased prothrombin time (PT) indicates the antithrombotic role of VD [1,11].

VD increases its antithrombotic role by reducing the production of plasminogen activator inhibitor-1 (PAI1), which is an inhibitor of plasminogen activator. Tissue plasminogen activator (TPA) is a tissue-type plasminogen activator, which is an endothelial enzyme that, by acting on plasminogen, converts it into plasmin [11]. In recent studies, high levels of PAI1 and an increase in the PAI1 to 2 ratio in pregnant women with VD less than 50 nmol/L have been observed. The role of PAI1 in thrombotic activities is already known; with increasing PAI1 level in the blood, it causes a lack of clearance of fibrin caused by clot formation, and, to the contrary, a reduction in its level reduces the risk of venous thrombosis and arterial dominance. The growth and development of the fetus will suffer as a result of increasing PAI1 as more fibrin precipitates with increased activity of this inhibitor, reducing blood flow between the mother and the fetus. Decreased levels of VD lead to complications in pregnancy, such as impaired fetal growth and development and pre-eclampsia. An increased ratio of PAI1 to 2 is also a measure of inappropriate placental function, which is high in low-VD pregnant mothers [12].

In a study conducted in 2020 on 899 patients, a negative correlation between the number of platelets and VD was observed; therefore, a reduction in VD increases the number of platelets. The patients in this study had no chronic or underlying diseases that affected platelet count [19]. In addition, changes in platelet count are also seasonal, as according to a study conducted on 36,644 adults and 27,478 children, platelet components and CRP are higher in winter, and this result is the opposite for RBC. This result was also obtained for platelets by the study of Gallerani et al. Therefore, it can be said that seasonal changes may play a role in reducing the incidence of thrombosis [20,21]. In addition to all of these cases, platelets are one of the important factors in clot formation, in which VDR has recently been found to play a role. In particular, VDR—which is a calcium flux regulator—plays an important role in the processes of megakaryopoiesis and platelet activation, which are important calcium-dependent events [1,14].

In addition to the roles of VD in thrombosis and thrombotic events, studies have reported the relationship of this vitamin with acute coronary syndrome (ACS), where low levels of VD were evident in these patients. Patients with reduced VD in coronary angiography were associated with more severe coronary artery disease (CAD) than normal subjects (in terms of VD) [22].

Despite all the above, in the relationship between VD and thrombosis, a study conducted under the title of Systemic Review in 2023 stated that most of the eligible studies have not found a significant relationship between hemostatic parameters and VD, with conflicting results between the parameters of thrombin production, and it has been found that VD inhibits tissue factor. Contradictory results between fibrinolytic parameters and VD were also reported in this study, and it was stated that studies with higher quality and better randomization should be performed to clarify this relationship [23]. In addition, studies have assessed the relationships between VD and brain trauma patients. This retrospective cohort study of 190 patients reported that low vitamin D levels in TBI patients were associated with acute DVT. There was a significant difference between those with vitamin D levels below 30 ng/mL and those above 30 ng/mL with *p* < 0.05, suggesting that an increased risk of DVT was associated with vitamin D levels below 30 ng/mL. Due to the change in mental state and dysphagia, these patients obtain less nutrients into their body: the more severe the disease, the higher the degree of nutrient deficiency and disorder and the higher the need for vitamin supplements. Immobility, high procoagulant states, and weight-bearing are among the factors that increase the risk of DVT thrombosis in these patients. The studies conducted point to the effects of VD in terms of improving the condition of brain trauma patients [24].

### 2.2. Vitamin D and Its Relationship with Inflammatory Pathways

VD has an anti-coagulant effect, which is mediated through suppressing inflammation and preventing endothelial damage and dysfunction, which act as factors in clot formation. A decrease in the VD level is associated with an increase in high-sensitivity C-reactive protein, which indicates inflammation, as well as an increase in the concentration of asymmetric dimethylarginine, which indicates functional impairment, endothelial damage, and increased risk of VTE [25].

**Figure 1:**
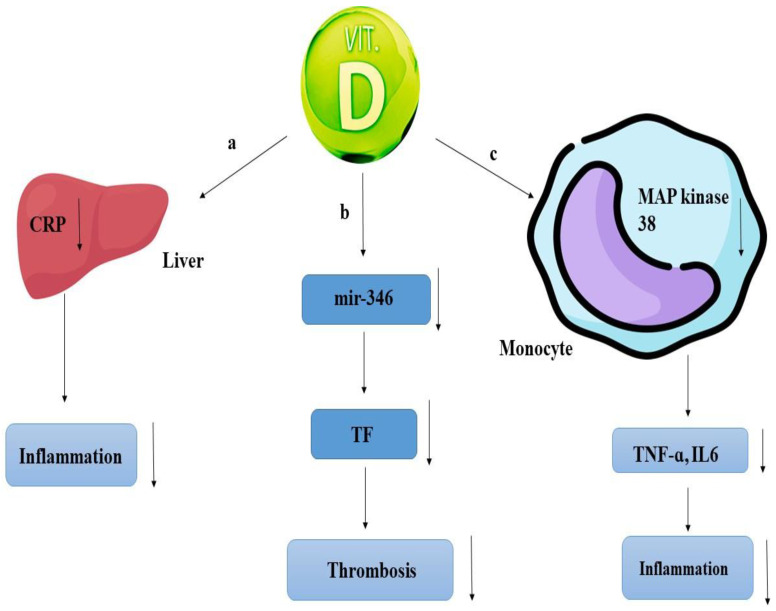
(a) Vitamin D can play a role in reducing inflammation through the reduction in C-reactive protein (CRP), which is an acute phase protein, subsequently leading to a reduction in inflammation; (b) Vitamin D plays a role in reducing the expression of tissue factor (TF) through reducing the expression of microRNA-346 (Mir-346), which will reduce thrombosis; (c) Vitamin D, through the MAP kinase 38 signaling pathway, reduces the expression of inflammatory factors IL6 and tumor necrosis factor α (TNF-α), subsequently reducing inflammation.

Tumor necrosis factor α (TNF-α) increases TF expression and thrombotic activities through the nuclear factor kappa B (NF-KB) pathway, and the TF promoter has a direct interaction with NF-kB. Studies have shown that procoagulant events occur under inflammatory conditions via increased expression of TF in smooth muscle cells, and Vitamin D can have potential beneficial effects under these conditions by regulating the pathway with the help of miR346, thus reducing the expression of TF (Figure 1) [26].

The connections and interactions between the coagulation system and innate immunity—with these two systems activating and helping each other—led to the coining of the term immunothrombosis in 2013 [1,27].

Studies have even shown that in the process of thrombosis, inflammation starts earlier than the coagulation system, which reveals the vital role of inflammation. In the process of thrombosis, the transcription of factors such as caspase 1, IL18, IL1 beta, and NLRP-3—which are considered inflammatory factors—is increased. There are connections between the second pyrin-containing NLRP-3 of the inflammatory complex and the second nucleotide binding of the leucine-rich family and hypoxia-inducible factor I in hypoxia-induced thrombosis [1,28]. In addition, when increasing the inflammatory activity of NLRP-3 and the release of IL1 beta in mice with low CD39, thrombosis returned to normal [1].

TF, which is an important factor for the activation of the external pathway of coagulation, is increased by inflammatory activity and its mediators; meanwhile, in the normal state of blood circulation, its amounts are low. Its main source is peripheral blood leukocyte cells [1,29].

Platelets, in addition to storing and releasing the materials needed for maintaining the integrity of blood vessels and thrombus, also carry cytokines, chemokines, and growth factors that affect immune responses [1,30]. The interactions between platelets and neutrophils, lymphocytes, and monocytes strengthen venous coagulation and lead to platelet activation [31]. In addition, the presence of lac receptors, such as toll-like receptors (TLR4, 9 and 2), on platelets establishes a stronger connection between immunity and thrombosis [32].

Fibrinogen is also an acute phase protein in tissue damage and inflammations, strengthening the bridge between inflammation and thrombosis [1,33].

The role of VD, as evident in thrombosis, is also evident in inflammation. This vitamin is known to play a role in inflammation by inhibiting the signaling of 38 map kinase. This signaling pathway causes the production of inflammatory cytokines such as TNF-ɑ and IL6. As a result, by inhibiting this path, the production of inflammatory factors will be prevented. VD with map kinase phosphatase 1 causes dephosphorylation of p38 and prevents its activation, as well as inhibiting cytokines such as TNF-α and IL6, which are produced IL10 by monocytes under lipopolysaccharides (LPS) inflammatory conditions. The dose of VD for this procedure should be above 30 ng/mL (Figure 1) [1,34].

Furthermore, studies have shown that by adding VD and incubating it with intestinal peripheral T cells in the context of inflammatory bowel diseases, due to a significant reduction in the signaling of T cell messages to produce interferon-gamma, TNF-ɑ, IL17, IL9, and IL22, the anti-inflammatory effects of VD were prominently observed [1,35].

In addition, this anti-inflammatory vitamin reduces the expression of the NF-kB signaling pathway by increasing the expression of IKBa [34,36].

Macrophage and dendritic cells also convert VD into its active form, for which they possess 25 hydroxylases and 1 α-hydroxylase enzyme, which demonstrates the importance of this vitamin in the inflammatory and immune systems [1,37].

Vitamin D in its active form, its binding protein, and the vitamin D receptor prevent T cell proliferation, and other actions of this vitamin include the inhibition of proliferative cytokines such as IFN-γ, IL2, and IL17 [38,39,40]. It also adds to its anti-inflammatory effects by creating IL10 and the growth of regulatory T cells. By inhibiting IL4 transcription, it can also reduce the effects of T helper 2 (Th2) cells. In general, inhibition of Th1 and Th17 responses regulates the growth of T cells, and controlling the localization of T-helpers is among the key functions of VD. It has also been shown that CD8 cells without VDR can reproduce without antigen [37].

Although VD deficiency and its relationships with infectious diseases have been the focus of many studies, the use of VD to treat these types of diseases is still debatable, with often contradictory results having been reported [41]. A randomized trial from December 2008 to March 2009 by Urashima et al. showed that taking vitamin D supplements in winter can reduce the incidence of influenza A among schoolchildren. The study was double-blind and compared vitamin D supplementation with a placebo [42]. Some studies have shown that this vitamin can reduce the symptoms, duration of treatment, and the amount of pathogens. In one study, the average infection score of patients who took vitamin D was 202, compared to 249 in the placebo group, which was significantly reduced with a 95% confidence interval and *p* = 0.04 [43]. However, one study stated that although the data on vitamin D in acute respiratory infections are promising, the results are not clear regarding COVID-19. It also stated that even increased mortality was observed in people who took vitamin D in high doses in patients with sepsis, respiratory distress syndrome, and infection. The baseline vitamin D in these patients was 11.2 ± 4.8 [44]. However, through the key and direct roles it has for immune cells, as mentioned above, VD may be relevant in the treatment of infectious and autoimmune diseases such as rheumatoid arthritis (RA), systemic lupus erythematosus (SLE), and multiple sclerosis (MS) [45].

Studies have also reported the effects of VD on CV health, with negative CV effects seen in patients with low levels of VD [46]. As mentioned above, VDR is an intracellular VD receptor, and, after binding to VD, it passes to the nucleus by joining the RxR (retinoid X receptor) and induces the production of VD-dependent proteins. This receptor has been found in macrophages, dendritic cells, and platelets, as well as the main types of cardiovascular cells, such as endothelial cells and cardiomyocytes, and VDR gene expression can induce and regulate CV health, while reducing its synthesis increases the effects of cardiovascular disease (CVD). A decrease in the expression of VDR has been reported in elderly people. Genetic polymorphisms occurring in VD binding protein (DBP) can lead to VD deficiency and increase the risk of CVD. Decreasing VD, in addition to increasing the mass of the left atrium, increasing the level of atrial natriuretic peptide, and causing an imbalance in homeostasis, causes fatty inflammation of the vessel wall and increases the risks of atherosclerosis and CVD, which has been investigated in humans and animals [46,47,48]. Additionally, VD can play an important role in reducing the risk of CVD by regulating the renin–angiotensin system, which suppresses smooth vascular muscles and improves vascular saturation, insulin resistance, and anticoagulant activity in general [49].

A non-linear Mendelian randomization analyses study also supports the finding that reduced vitamin D levels increase CVD risk [9].

Studies have also reported a significant relationship between the homocysteine level and the rate and prevalence of CAD in patients with VD deficiency. Hypercysteinemia often occurs in chronic kidney disease patients, which is related to kidney dysfunction leading to the incomplete excretion of homocysteine metabolites. This situation can lead to endothelial cell dysfunction, platelet activation, and prothrombotic conditions, all of which cause cardiovascular events and strokes [50].

In addition, studies have shown that a lower prevalence of myocardial infarction is associated with vitamin D levels above 30 ng, such that there is a lower incidence of myocardial infarction at levels above 30 ng compared to the range of 21 to 29 and below 20 ng of vitamin D [51].

A study showed that vitamin D levels were significantly associated with all-cause mortality. During 14 years of follow-up, 18,700 of the 307,601 participants died. A significant reduction in the risk of death was shown with increasing vitamin D concentrations to 50 nanomoles per liter. The recorded deaths were from all causes, including CVD, cancer, and respiratory diseases [52].

### 2.3. Vitamin D and Its Relationship with Endothelial Cells

Maintaining vascular integrity and transporting solutes and nutrients through the inner layer of the vessel is one of the functions of endothelial cells. Prothrombic or antithrombic conditions depend on the activation and inactivation of these cells [1]. When activated by TF expression and thrombin production, they play a prothrombic role [53]. Different factors, such as endothelin 1, P-selectin, angiopoietin 2, and VWF, are involved in platelet adhesion, inflammation, and vasoconstriction, all of which are produced by activated endothelial cells [54,55]. Conversely, in conditions of endothelial cell inactivation, the coagulation cascade and platelet adhesion are inhibited, preventing clot formation [56].

Endothelial progenitor cell receptors, TFPI thrombomodulin, and heparin-like proteoglycans are anticoagulant factors that are associated with endothelial cells. [53]. In one study, VD supplementation reduced endothelial dysfunction in CVD by increasing the level of nitric oxide (NO) [57].

In another study, 12 weeks of VD treatment improved endothelial function in patients with lupus erythematosus, whose endothelial function was impaired by VD deficiency. The reduction in VDR endothelial cells was associated with this disorder, with a molecular dependency. Increases in inflammatory cytokines such as IL6 and interferon-gamma, as well as high levels of CRP, were observed in VD-deficient patients [58].

On the other hand, considering that VD plays a role in reducing total body cholesterol, low-density lipoprotein (LDL), and triglyceride levels and increasing high-density lipoprotein (HDL), VD can be more strongly related to CVD and atherosclerosis. With an increase in selectin adhesion molecules as a result of the reduction in this vitamin, atheroma plaque can form more easily [59].

Further studies have shown the favorable effects on diastolic blood pressure level and parathyroid hormone concentration achieved in patients taking VD supplements. In a study of 299 patients, VD was suggested as an adjunctive therapy for coronary artery disease patients with VD deficiency [60].

VD plays a role in the anti-inflammatory pathway by increasing the expression of the IL10 receptor and inhibiting the activation of the NF-kB signaling pathway, as well as IL6 reducing the oxidative stress of cells. Considering the greater prevalence of decreased VD in women, VD plays a mediating role in the relationship between gender and DVT after stroke [13,61].

Another study, conducted in 2014 by Inteziri Maleki et al., investigated the level of VD and its relationships with Pue-selectin, hs-CRP, and thrombosis factors in VTE patients. Their study population was Iranians, and 25 hydroxy VD was used to measure VD levels. In their results, P-selectin was high as a diagnostic marker in VTE, and the value of hs-CRP was also reported above 10 mg/L, which indicated that the presence of inflammation can be a factor triggering thrombosis. hs-CRP levels were reduced by anticoagulation treatment in atrial fibrillation patients. Their previous studies had stated the relationships between VD reduction and age, high blood pressure, and vascular and cardiac disorders; however, in this study, no correlation between VD levels, P-selectin, and hs-CRP in VTE patients was observed [62].

The reduction in VDR as a result of the reduction in VD in monocyte cells leads to the creation of pro-inflammatory monocytes, which can increase platelet aggregation and activation, as well as increasing adhesion to endothelial cells, indicating a thrombotic event and atherosclerosis [63].

### 2.4. Vitamin D Deficiency and Oxidative Stress

The role of VD in improving the function of endothelial cells by reducing the production of inflammatory cytokines and increasing the production of NO—which, in turn, causes a reduction in platelet aggregation—has been shown in various studies.

**Figure 2:**
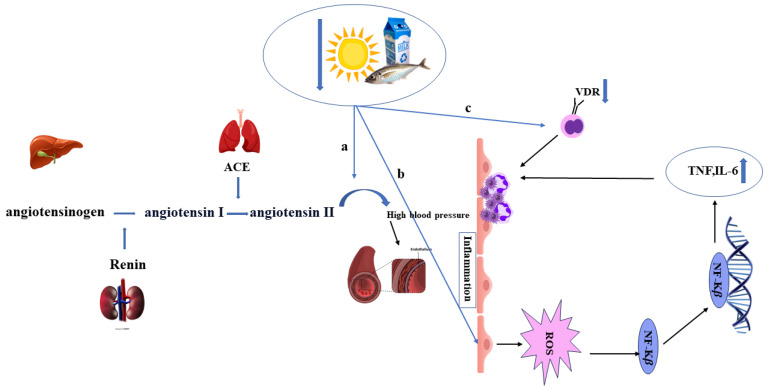
(a) Vitamin D deficiency leads to overexpression of renin and, hence, enactment of the RAS, leading to renal and cardiovascular injuries; (b) Nuclear factor-kappa B (NF-kB) plays a key role in leukocyte adhesion and inflammation by regulating endothelial cells and is inversely affected by vitamin D; (c) a decrease in vitamin D receptor (VDR) due to low vitamin D in monocytes leads to inflammatory cells promoting blood clotting and artery hardening.

NF-kB plays a significant role in the adhesion of monocytes, neutrophils, and lymphocytes to the vessel wall and the inflammation process, and, by activating and regulating endothelial cells, it is involved in prothrombotic activities. Notably, the signaling pathway of NF-kB is reduced by VD (Figure 2b). The expression of VDR and alpha-hydroxylase in endothelial cells reflects a special regulatory relationship of VD in endothelial cells, which increases NO production via VDR and decreases NO production if VDR is inactivated. Endothelial nitric oxide synthase (eNOS) upregulates NO, which has been shown in studies of eNOS depletion following VDR depletion [1,64].

Additionally, in a comparison between patients with sufficient and insufficient VD and reduced expression levels of p65 NF-kB, pro-inflammatory cytokines downstream of NF-kB were elevated in those with VD deficiency. In other studies, the reverse relationship between VD levels and the renin–angiotensin–aldosterone system has been proven; namely, this system is suppressed by VDR activation, which leads to improved blood pressure (Figure 2a).

Angiopoietin 2 causes vascular damage by activating NFKB and inducing the expression of several cytokines, including TNF-α and IL6, as well as the production of active species O_2_, which VD reduces via the mechanism of the angiopoietin peroxisome-activated receptor gamma receptor pathway [1,50].

Under normal conditions, ROS, the oxidative stress species—including superoxide anion O_2_^•−^ radicals and hydrogen peroxidase—play an important role in homeostasis. However, the creation of oxidative conditions and the rise of ROS under conditions such as obesity and diabetes can have negative effects on the body’s physiology. In addition to these cases, observational studies have shown the relationship between natural VD and the reduction in oxidative stress, and, vice versa, through chemical links between oxidative stress and VD, studies have reported the improvement of oxidative stress levels via VD treatment; furthermore, VD can reduce the level of oxidative stress species by increasing production of the GSH enzyme, which is an antioxidant. VD also helped to lower the levels of pro-inflammatory factors in diabetic mice, which was shown by observing the effects of VD in diabetic mice [49,64].

A systematic study with 820 participants found that VD and VDR improve endothelial function. Endothelial dysfunction and cardiovascular complications in CVD are caused by increased oxidative stress and inhibition of NO synthesis. The authors of the study used a marker called flow-mediated dilation (FMD) to measure the ability to produce nitric oxide by the endothelium and stated that with a 1% increase in this marker, cardiovascular events decreased by 8–13%. They considered VD as a mediator of the production of NO [65]. A study suggests 6.5% as a cut-off for optimal endothelial function [66].

The triangle of hypovitaminosis D, thickening of common carotid intima, and increased levels of oxidative stress are considered indicative of CV dysfunction; however, more studies are needed to clarify the relationships between VD and oxidative stress, such that VD dosages and durations of treatment providing definite benefits can be used in the context of CVD treatment [22].

In one study, VD supplementation reduced endothelial dysfunction in CVD by increasing the level of NO; in other words, with an increase in the level of oxidative stress, the dilation of skin vessels is reduced by nitric oxide, which can be improved with VD [57].

After 12 weeks of VD treatment, endothelial function was improved in patients with lupus erythematosus, whose endothelial function was initially impaired due to VD deficiency. The reduction in VDR in endothelial cells was associated with this disorder, with a molecular dependency. Increases in inflammatory cytokines, such as IL6, interferon-gamma, and high levels of CRP, are typically observed in cases of VD deficiency [67].

## 3. Gender, Age, and Other Risk Factors Related to the Links Between Vitamin D Status and Thrombosis

The incidence of DVT has been observed to differ based on age and gender. Women under the age of 50 exhibit a lower incidence, while those over 65 have a significantly higher rate of DVT, with elderly females demonstrating a greater risk compared to their male counterparts [7]. Additionally, earlier research involving stroke patients indicated that females are at a higher risk for DVT than males [63,68]. Reasons for the increased risk of DVT in females may include several factors. Conditions such as pregnancy and estrogen antagonist therapy could explain the gender disparity in DVT, although these factors are not common among elderly stroke patients [69,70,71,72]. Other potential contributing factors include hormone replacement therapy, the hormonal changes associated with menopause [73], and the use of oral contraceptives (OCPs) [74].

According to a retrospective cohort study, up to 43.9% of elderly women were found to have insufficient VD [75]. Another study has revealed that VD deficiency is more prevalent among elderly, middle-aged, and perimenopausal women [76,77,78]. This may be attributed to factors such as differences in dietary intake according to gender [72], reduced exposure to sunlight [79], and the loss of estrogen [80].

The exposure of women to the sun (or even artificial UVB rays) can improve VD levels, with this increase in Vitamin D level consequently reducing the risk of VTE. Meanwhile, cold weather or other factors leading to less hours of exposure to the sun has the opposite effect [81]. Lower serum VD levels lead to decreased calcium absorption and a higher prevalence of osteoporosis, particularly among perimenopausal women [76].

Prior research has indicated notable gender differences in dietary VD intake; women tend to consume less VD during the winter and autumn months, according to a nationwide nutritional survey [82]. Furthermore, variations in body fat percentage between genders may also play a role in VD deficiency. Research has shown that a higher fat mass is linked to VD deficiency in women [83]. A separate study comparing DVT and non-DVT groups found that a greater percentage of females were present in the DVT group when compared to the non-DVT group. Additionally, fewer patients in the DVT group had sufficient VD levels, while there were higher prevalences of insufficiency and deficiency. There were no notable associations between VD level and age, BMI, Padua score, or D-dimer levels; however, a marginally significant relationship was observed between VD level and the National Institutes of Health Stroke Scale (NIHSS) score upon admission. Females exhibited lower VD levels and higher DVT rates than males, along with greater percentages of VD insufficiency and deficiency. This previous study highlighted three key points: First, females exhibit lower Vitamin D levels and a higher risk of DVT, when compared to males. Second, elevated VD levels correlate with a reduced risk of DVT. Third, VD may mediate the relationship between gender and DVT risk [61].

VD deficiency is more likely to occur in those with antiphospholipid syndrome than in normal people, and people who have antiphospholipid syndrome and Vitamin D deficiency are more prone to thrombotic events. Although VD deficiency is related to changes in latitude due to variations in the availability of UVB rays—which is one of the factors required for VD synthesis—this factor and avoiding sunlight are not among the causes of VD deficiency in those with antiphospholipid syndrome. Genetic polymorphisms including 25 hydroxylases, 1 ɑ-hydroxylase, 24 hydroxylases, D binding protein (DBP), and VDR genes are related to the reduction in VD levels. Additionally, the lack of this vitamin can contribute to autoimmune disorders in people with antiphospholipid syndrome [84].

**Figure 3:**
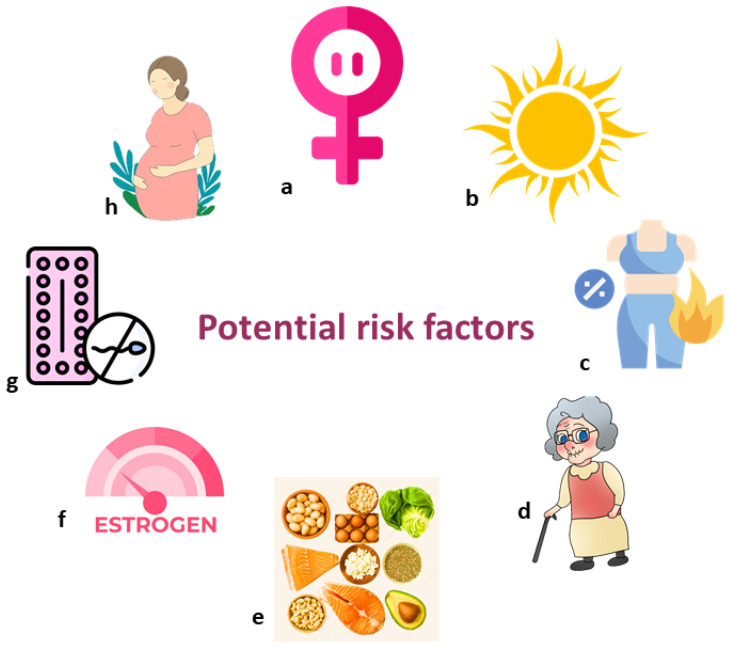
Potential factors that may contribute to the higher risk of thrombosis and vitamin D deficiency in women: (**a**) Menopause; (**b**) Less exposure to sunlight; (**c**) Higher body fat; (**d**) Age over 65; (**e**) Less dietary intake of vitamin D; (**f**) Loss of estrogen and estrogen antagonist therapy; (**g**) The use of oral contraceptives; (**h**) Pregnancy.

In summary, female stroke patients show a higher propensity for thrombosis—particularly DVT—when compared to males, with VD potentially playing a crucial role in this association. More research is necessary to determine whether VD supplementation can effectively lower DVT risk in stroke patients, particularly in women (Figure 3).

## 4. Discussion

Our study reviewed the current evidence regarding the relationships between VD levels and thrombosis, and our findings suggest that adequate VD levels can play a protective role against thrombotic events through vascular health and inflammation.

The literature indicates an association between low VD levels and an increased risk of thrombosis; as such, VD supplementation may reduce the risk of venous thromboembolism. A meta-analysis of multiple studies concluded that individuals with low serum VD levels had a significantly increased risk of VTE, compared to those with normal levels. There was significant heterogeneity among the studies, and low VD levels were positively associated with an increased risk of VTE in population-based studies and subjects below 60 years old [22].

A retrospective chart review analyzed 181 patients with VTE from July 2018 to June 2020, focusing on a subgroup of 110 patients with documented VD levels. Regarding VD levels, an alarming 85.7% of the patients with unprovoked DVT exhibited low VD levels (defined as less than 30 ng/mL); however, no significant correlation was found between VD levels and cancer-associated thrombosis. These findings suggest that VD deficiency might have served as a risk factor for unprovoked VTE, indicating an inverse relationship with the VD level [63].

In a study conducted by Xiang et al., the association between vitamin D and the risk of venous thromboembolism in diabetic subjects was assessed. They reported 10,645 VTE cases among 378,822 participants during a 12.5-year follow-up period. They reported that there was a significantly stronger inverse association between serum vitamin D levels and the occurrence of VTE in diabetic subjects. This was also found for PE and DVT. Despite the finding of a strong inverse association between serum vitamin D levels and incident VTE, the VDR rs22228570AA genotype among diabetic subjects did not significantly modify the association between incident VTE and serum vitamin D levels. Consequently, it can be said that in diabetic subjects, vitamin D levels are inversely associated with VTE risk regardless of genetic risk factors for VTE [85].

A retrospective cohort study that analyzed VD levels in a population of 190 traumatic brain injury (TBI) patients upon admission demonstrated the relationship between VD levels and the occurrence of acute DVT in patients with TBI during acute inpatient rehabilitation. The findings revealed that 62% of these patients had low VD levels. Among those with low VD, 21% developed acute DVT during their rehabilitation stay, compared to only 8% of patients with normal VD levels. The study concluded that VD levels below 30 ng/mL are associated with an increased likelihood of acute DVT in individuals with moderate to severe TBI, highlighting the potential role of VD in thromboembolic risk within this population [24].

Epidemiological studies have indicated that patients with cardiovascular conditions frequently exhibit low levels of 25(OH)D (below 20 nm/mL), while increased VD levels correlate with a lower risk of venous thromboembolism. Additionally, the active variant of VD—1,25-dihydroxy VD—has been shown to promote the inhibition of blood coagulation, prompting inquiries into its possible protective function against thrombosis [1,2].

The review study conducted by Shubham Khanolkar et al. in 2023 demonstrated that maintaining adequate VD levels can positively influence inflammation, immune response, endothelial function, lipid metabolism, and vascular health, thereby affecting the prevention of atherosclerosis and heart disease [86]. Prospective studies conducted by P. Brøndum-Jacobsen et al. within the Copenhagen City Heart Study and the Copenhagen General Population Study, which involved 18,791 participants from Copenhagen, Denmark, across various age groups and conducted over several decades, revealed a significant association between low plasma levels of 25-hydroxyvitamin D and an increased risk of thromboembolism. Participants in the lowest VD tertile presented a 37% increased risk, which rose to 70–103% following adjustments for confounding factors and regression dilution bias [87].

A study analyzed data from 3316 patients in the LURIC study who underwent coronary angiography between 1997 and 2000. The levels of 25(OH)D and 1,25(OH)2D were measured, with adjustments made for seasonal variations. Over a follow-up period of 7.75 years, 769 patients died, including 42 from fatal strokes. The findings indicated that low levels of both types of VD were significantly associated with an increased risk of fatal stroke, suggesting the potential benefits of VD supplementation for stroke prevention [88]. A clinical trial demonstrated that calcitriol could lower rates of both venous and arterial thrombosis in cancer patients [89].

The study conducted by Almeida Moreira Leal et al. in 2020 aimed to explore the molecular mechanisms of VD3 and its effects, both centrally and peripherally. Mice treated with VD3 for seven days underwent assessments for anti-inflammatory and antinociceptive activities using models of acute pain and inflammation. The results demonstrated that VD3 significantly reduced pain and inflammation, as indicated by decreased swelling, lower inflammatory cell counts, and reduced TNF-alpha expression. Additionally, VD3 inhibited the production of inducible nitric oxide synthase (iNOS) and COX-2 in the brain and lessened neutrophil degranulation by decreasing reactive oxygen species (ROS) and myeloperoxidase (MPO) release. This study emphasized the strong anti-inflammatory and antioxidant effects of VD3 in different parts of the body [90].

In observational studies, VD deficiency has been linked to an elevated risk of future arterial CVD and potentially VTE [72,87,91]. In contrast, interventional studies on VD did not demonstrate a protective role of vitamin D [92,93,94].

There is no consensus on the best level of serum 25(OH)D level, but most specialists classify vitamin D deficiency as a level below 20 ng/mL and insufficiency as ranging from 21 to 29 ng/mL. Therefore, research indicates that the optimal concentration should be at least 30 ng/mL [95].

Another extensive observational study that tracked 247,574 adults from Denmark for 0–7 years revealed that both low levels of 25(OH)D (approximately 12.5 nmol/L [5 ng/mL]) and high levels (approximately 125 nmol/L [50 ng/mL]) were linked to an increased risk of mortality from CVD, stroke, and acute myocardial infarction [96]. High serum cholesterol and hypertension are two primary risk factors for CVD. The findings on supplemental vitamin D and cholesterol levels are varied, as demonstrated in a meta-analysis of 41 clinical trials involving 3434 participants (average age 55 years). This analysis revealed that vitamin D supplementation ranging from 0.5 mcg (20 IU) to 214 mcg (8570 IU)/day (for an average of 2795 IU) with durations ranging between 6 weeks and 3 years led to reductions in serum total cholesterol, low-density lipoprotein cholesterol, and triglyceride levels but did not affect high-density lipoprotein cholesterol levels [97].

Doctors ought to contemplate testing vitamin D levels in patients who are susceptible to thrombosis through the 25-hydroxyvitamin D test (25(OH)D test). This test assesses the amount of vitamin D in the blood and aids in detecting deficiencies that may heighten the risk of thrombotic occurrences [98]. When a patient is identified as being at risk, the 25(OH)D test must be requested to assess serum 25-hydroxyvitamin D levels. The results should be analyzed thoughtfully: levels lower than 30 nmol/L (12 ng/mL) signify a deficiency, whereas levels ranging from 50 to 125 nmol/L (20 to 50 ng/mL) are deemed adequate [62,98]. Certain groups are particularly likely to develop thrombotic events. These groups include older individuals, people with extended immobility due to bed rest or lengthy travel, those undergoing significant surgical procedures, and patients with chronic conditions such as heart disease, lung disease, or cancer. Elevated estrogen levels due to the use of oral contraceptives or hormone replacement therapy, obesity, a family history of blood clots, smoking, or an unhealthy diet also heighten the risk [62].

Due to the widespread occurrence of VD insufficiency globally—particularly in groups with limited sunlight exposure—it is crucial to develop methods to enhance VD levels. This might involve changing diet, taking supplements, or promoting safe sun exposure habits through public health initiatives. Correcting VD deficiencies could potentially lower the occurrence of blood clotting events, thus decreasing the morbidity and mortality associated with cardiovascular disease. Additional research is required to uncover the precise ways in which vitamin D impacts thrombosis. Studying genetic components that could impact how individuals react to VD may also yield beneficial findings. Moreover, studying how VD interacts with other nutrients in the context of thrombotic risk will enhance our comprehension of this intricate relationship.

## 5. Conclusions

Given the prevalence of VD insufficiency, particularly among populations with limited exposure to sunlight, this research highlighted the urgent need for strategies to increase VD levels through dietary modifications and supplementation. Correcting VD deficiencies may mitigate the morbidity and mortality associated with cardiovascular disease by reducing the frequency of thrombotic events. More research is needed to decipher the complex interactions between vitamin D, genetic factors, and lifestyle choices, with the aim of improving clinical approaches for the prevention and management of thrombosis.

## Abbreviations

VD	Vitamin D
VTE	Venous thromboembolism
DVT	Deep vein thrombosis
PE	Pulmonary embolism
CV	Cardiovascular
hs-CRP	High-sensitivity C-reactive protein
VWF	Von Willebrand factor
TF	Tissue factor
VDR	Vitamin D receptor
TFPI	Tissue factor pathway inhibitor
PT	Prothrombin time
PAI-1	Plasminogen activator inhibitor 1
TPA	Tissue plasminogen activator
ACS	Acute coronary syndrome
CAD	Coronary artery disease
MPV	Mean platelet volume
ED	Erectile dysfunction
NF-Kβ	Nuclear factor-kappa β
TLR	Toll-like receptor
TNF-ɑ	Tumor necrosis factor ɑ
LPS	Lipopolysaccharides
TH	T helper
RA	Rheumatoid arthritis
SLE	Systemic lupus erythematosus
MS	Multiple sclerosis
RXR	Retinoid X receptor
CVD	Cardiovascular disease
DBP	VD binding protein
IL	Interleukin
Mir	Micro RNA
NO	Nitric oxide
HDL	High-density lipoprotein
LDL	Low-density lipoprotein
eNOS	Endothelial nitric oxide synthase
iNOS	Inducible nitric oxide synthase
FMD	Flow-mediated dilation
OCPs	Oral contraceptives
NIHSS	National Institutes of Health Stroke Scale
TBI	Traumatic brain injury
ROS	Reactive oxygen injury
MPO	Myeloperoxidase
